# Artesunate Abolishes Germinal Center B Cells and Inhibits Autoimmune Arthritis

**DOI:** 10.1371/journal.pone.0104762

**Published:** 2014-08-12

**Authors:** Lifei Hou, Katharine E. Block, Haochu Huang

**Affiliations:** 1 Department of Medicine and Knapp Center for Lupus and Immunology Research, University of Chicago, Chicago, Illinois, United States of America; 2 Committee on Immunology, University of Chicago, Chicago, Illinois, United States of America; The Ohio State University, United States of America

## Abstract

The antimalarial drug artemisinin and its derivatives exhibit potent immunosuppressive activity in several autoimmune disease models, however the mechanisms are not well-understood. This study was designed to investigate the therapeutic effects and the underlying mechanisms of the artemisinin analog artesunate using the K/BxN mouse model of rheumatoid arthritis. The well-studied disease mechanisms of K/BxN model allowed us to pinpoint the effect of artesunate on disease. Artesunate treatment prevented arthritis development in young K/BxN mice by inhibiting germinal center (GC) formation and production of autoantibodies. In adult K/BxN mice with established arthritis, artesunate diminished GC B cells in a few days. However, artesunate did not affect the follicular helper T cells (Tfh). In contrast to the spontaneous K/BxN model, artesunate treatment exerted minor influence on K/BxN serum transfer induced arthritis suggesting that artesunate has minimal effect on inflammatory responses downstream of antibody production. Finally, we showed that artesunate preferentially inhibits proliferating GC B cells. These results identify GC B cells as a target of artesunate and provide a new rationale for using artemisinin analogues to treat autoimmune diseases mediated by autoantibodies.

## Introduction

Artemisinin is sesquiterpene endoperoxide produced by the plant *Artemisia annua L* [1]–[3]. It has long been used in traditional Chinese medicine to treat malaria. Artemisinin and its derivatives have been recommended by World Health Organization as first-line treatment of multidrug-resistant malaria worldwide. Artemisinin inhibits specifically and selectively the sarco/endoplasmic reticulum calcium ATPase of malaria parasite *Plasmodium falciparum* [4]. Besides having antimalarial activity, artemisinin and its derivatives also exhibit potent immunosuppressive activity in several autoimmune disease models, including systemic lupus erythematosus [5]–[7], rheumatoid arthritis [8]–[11], and experimental autoimmune encephalomyelitis [12], [13]. Although the underlying mechanisms are not well-understood, artemisnin can inhibit the proliferation and differentiation of T cells [5], [14], [15]. Artemisinin analogs have been shown to suppress the differentiation of both Th1 and Th17 cells and induce the development of Treg cells *in vitro* and *in vivo* [6], [10], [13], [16]. In addition, artemisinin analogues, such as artesunate, were reported to directly suppress the proinflammatory cytokine production in the autoimmune diseases [9], [17]. However, the effect of artemisinin analogues on B cells, especially the germinal center (GC) response in autoimmunity, is not known.

B cells are central players in the adaptive immune response, undergoing activation and further differentiation into plasma or memory cells in GCs. In many autoimmune diseases, B cells are often major drivers of the autoimmune response through production of autoantibodies, and other functions such as antigen presentation and cytokine or chemokine production [18]–[21]. Thus, although the major clinical immunosuppressive agents are targeting T cell activation, there is increasing interest in treating autoimmune diseases by depleting B cells or suppressing B cell survival with drugs such as rituximab (anti-CD20 mAb) and belimumab (anti-BAFF mAbs) [18], [22].

The GC is a highly dynamic microenvironment within B cell follicles of secondary and tertiary lymphoid organs where antigen-activated B cells rapidly expand and differentiate, and then generate plasma cells that produce high affinity antibodies [23]. The process is critically dependent on the intimate interaction of GC B cells and follicular helper (Tfh) T cells. Tfh cells are a distinct T cell subset controlled by transcription factor Bcl6 [24]–[26]. They are characterized by the expression of chemokine receptors required for migration to B cell follicles (downregulation of CCR7 and upregulation of CXCR5) as well as expression of surface molecules involved in cell-cell interaction (PD-1, ICOS, SAP) and cytokine production (IL-4 and IL-21) [27]. Tfh cells are important for generating protective antibodies, but dysregulation of Tfh cells can also drive self-reactive B cells to produce autoantibodies in autoimmune diseases.

In the current study, we investigated the effect of an artemisinin analogue, artesunate, in the K/BxN mouse model of rheumatoid arthritis. K/BxN mice spontaneously develop an autoimmune inflammatory disease with many clinical, histopathological and immunological features of the human disorder [28], [29]. Breakdown of T and B cell tolerance leads to the production of high-titer autoantibodies against glucose-6-phosphate isomerase (GPI), which directly induces joint pathology [30]. Given the well-studied disease mechanisms and clearly defined roles of various immune cells, K/BxN mice have been an informative model to investigate therapeutic agents targeting antibody-mediated autoimmune diseases.

We found that artesunate prevented the development of arthritis in young K/BxN mice by inhibiting differentiation of GC B cells and production of autoantibodies. However, artesunate did not significantly affect Tfh cells or the inflammatory phase of the disease. Furthermore, we showed that artesunate suppresses the proliferation of GC B cells, an extremely proliferative population among immune cells. Collectively, our findings reveal a new target of the immunosuppressive functions of artesunate and have implications in the clinical application of artemisinin analogues to treat antibody-mediated autoimmune diseases.

## Materials and Methods

### Mice

K/BxN mice were generated by crossing B6.KRN TCR transgenic mice to NOD mice. All mice were housed in specific pathogen free facility at University of Chicago. All experiments in this study were approved by the Institutional Animal Care and Use Committee of the University of Chicago (Protocol 71847). All animals were euthanized via CO_2_ followed by cervical dislocation.

### Artesunate treatment

Both preventive and therapeutic treatments were done on K/BxN mice. In preventive treatment, 3-week-old mice were randomly divided into vehicle control group and artesunate group and were treated (i.p., 100 mg/kg, twice/day) for 2 weeks. In therapeutic treatment, 6-week-old mice were treated with vehicle or artesunate for 2 weeks (i.p., 100 mg/kg, twice/day) or 2 months (i.p., 100 mg/kg, once/day). Ankle thickness was monitored every day. After treatment, individuals were euthanized and sera and spleens were collected. Artesunate (Sigma-Aldrich) was initially dissolved in DMSO or 5% NaHCO3 and adjusted in PBS. Vehicle was prepared in the same way without artesunate.

### K/BxN serum-induced arthritis

Adult B6 mice were injected i.p. with K/BxN serum at day 1 and day 3 (150 ul/mouse/injection). Mice were treated with vehicle or artesunate for 15 consecutive days from day 0 to day 14 (i.p., 100 mg/kg, twice/day). Ankle thickness was monitored every day.

### Immunohistochemistry

Spleens of treated mice were embedded in OCT compound and snap-frozen at −70°C. Cryosections of spleen (6 µm) were stained with FITC-labeled PNA, PE-labeled anti-mIgD, and APC-labeled anti-KRN (clone 3-4G-B7) antibodies.

### Flow cytometric analysis

Various markers for Tfh cells and GC B cells and intracellular Bcl-6 and Foxp3 staining was conducted and analyzed as described [31]. For intracellular cytokine staining, cells were incubated for 4 hours with phorbol 12-myristate 13-acetate (PMA, 50 ng/ml) and ionomycin (750 ng/ml) in the presence of BFA (10 µg/ml). At the end of incubation, suspended cells were collected, blocked with FcγR antibody before surface staining with fluorescence-labeled antibodies. After surface staining, cells were fixed, permeabilized, and stained for cytokines according to manufacturer's instruction (BD Cytofix/Cytoperm kit).

### ELISA

Serum titers of anti-GPI IgG were determined by ELISA. Plates were coated with 5 µg/mL of recombinant mouse GPI. After incubating with serial dilutions of serum samples, bound antibodies were detected by adding biotinylated goat anti-mouse IgG (subclasses 1+2a+2b+3) Fcγ fragment-specific antibody (Jackson ImmunoResearch Laboratories Inc.) followed by alkaline phosphatase-conjugated streptavidin. The data were fitted by a four-parameter curve using Prism software (GraphPad). Titer is defined as the serum dilution that gave an optical density of 50% maximum (inflection point) of the curve.

### Statistical analysis

Data were analyzed using Student's t-test or 2-way ANOVA. P<0.05 was considered to be statistically significant.

## Results

### Artesunate prevents the development of arthritis in K/BxN mice

**Figure 1:**
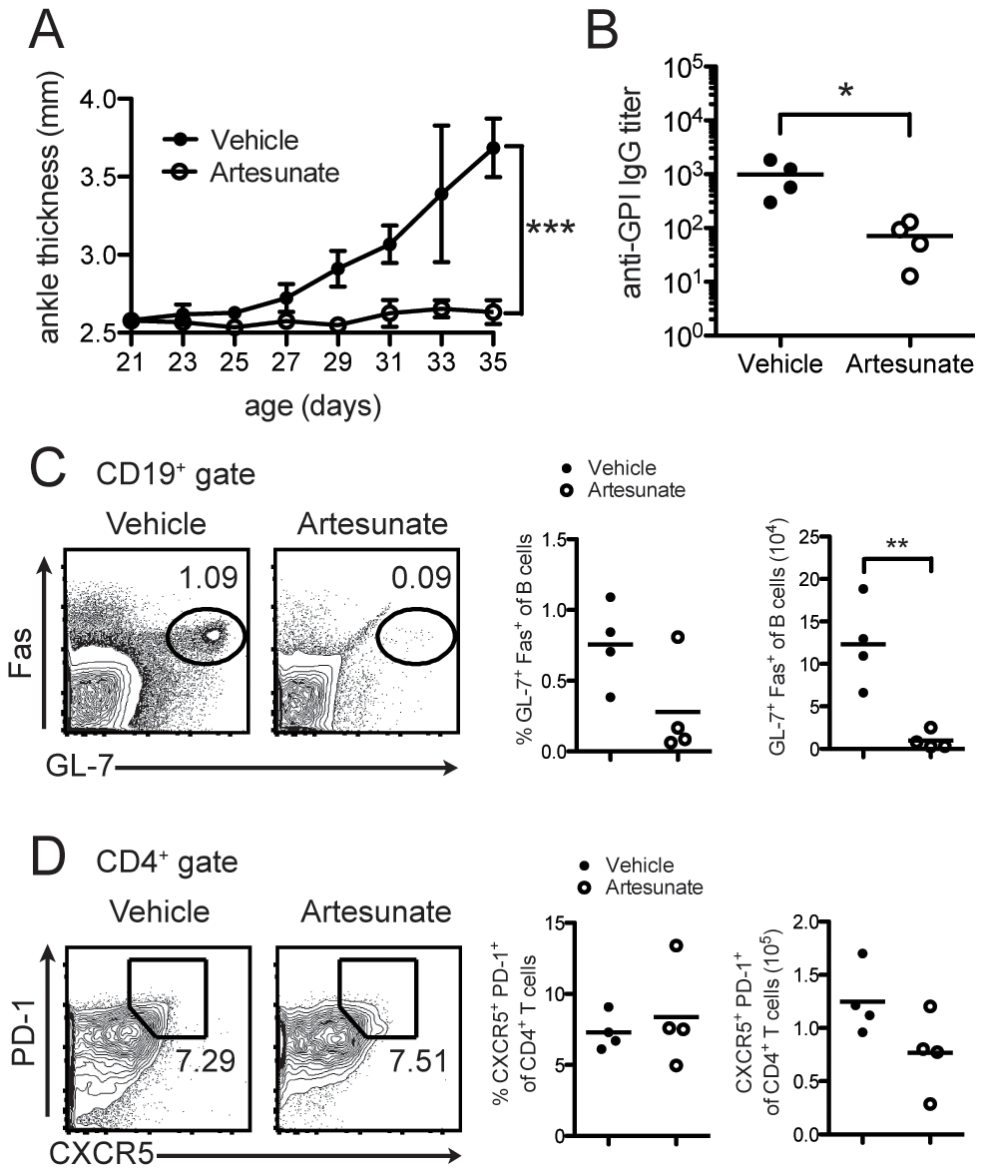
Artesunate treatment of young K/BxN mice. (**A**) Ankle thickness of 3-week old K/BxN mice were treated with artesunate for 2 weeks (100 mg/kg, twice/day). Ankle thickness is shown as mean ± SD (n = 4 per group); (**B**) serum anti-GPI titers of mice treated in (A) at 5 weeks of age, each symbol represents one mouse; (**C**) flow cytometric analysis of GC B cells by Fas and GL-7 staining; (**D**) flow cytometric analysis of Tfh cells by PD-1 and CXCR5 staining at 5 weeks. Representative of two experiments. ***  = p<0.0001 (2-way ANOVA, (A)); *  = p<0.05, **  = p<0.01 (Students t-test, (B–C)).

K/BxN mice develop arthritis spontaneously by 4 weeks of age. To test the effects of artesunate on arthritis development, we treated 3-week old K/BxN mice with artesunate twice a day at a dose of 100 mg/kg body weight. This dose is similar to what have been used to treat malaria clinically. As shown in Fig. 1A, vehicle treated mice developed arthritis around 28 days with maximum disease severity at 35 days of age. In contrast, artesunate treatment inhibited arthritis completely in 80% of the mice. The pathogenesis of K/BxN arthritis depends on both adaptive and innate immune response and can be divided into the inductive phase where autoreactive T and B cells are activated to produce autoantibody, and the effector phase where immune complexes trigger complement activation and recruit innate immune cells, leading to joint destruction [32]. To determine the influence of artesunate on the inductive phase, we examined serum anti-GPI IgG levels, and the differentiation of T follicular helper cells (Tfh) and GC B cells. Serum anti-GPI IgG antibody titer in artesunate treated mice was decreased by one order of magnitude compared to vehicle treated mice (Fig. 1B). Flow cytometric analysis demonstrated that the GC B cells were largely absent in artesunate treated mice (Fig. 1C), consistent with the decrease of anti-GPI antibody titers. However, the CXCR5^+^PD-1^hi^ Tfh cells were comparable in both vehicle- and artesunate-treated mice in both the percentage and the total numbers (Fig. 1D). These data suggest that artesunate affects the adaptive response by inhibiting GC B cell differentiation.

### Artesunate has a minor effect on the effector phase of disease

**Figure 2:**
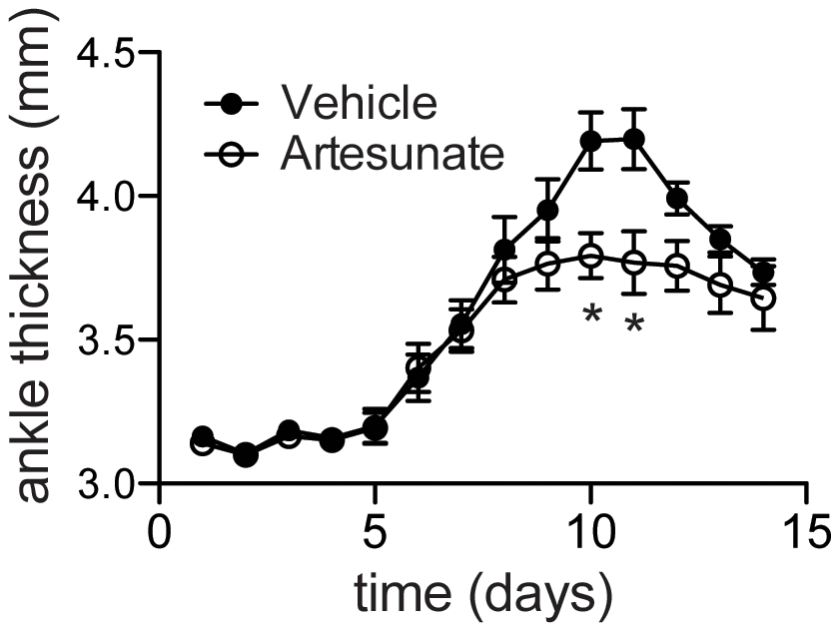
Effect of artesunate on serum transfer-induced arthritis. Six-week old B6 mice were injected i.p. with 150 ul K/BxN serum at day 1 and 3. Artesunate and vehicle control were treated from day 0 to day 14. Ankle thickness is shown as mean ± SD (n = 4 to 5 per group). Representative of two experiments. *, p<0.05 for the two indicated time-points.

Transfer of serum or purified antibodies from arthritic K/BxN mice into healthy animals provokes arthritis quickly and robustly [32]. This allowed us to test the effect of artesunate on the effector phase independent of the inductive phase. As shown in Fig. 2, recipient mice began to develop arthritis within days after serum transfer and the disease peaked around 10 days. Artesunate treatment had no effect on disease onset but showed a minor amelioration of the ankle swelling at day 10 and day 11 after the serum transfer. Thus, we conclude that artesunate primarily suppressed the adaptive immune response to diminish the autoantibody production rather than inhibiting innate inflammatory responses in K/BxN mice.

### Artesunate suppresses the GC response in mice with established arthritis

**Figure 3:**
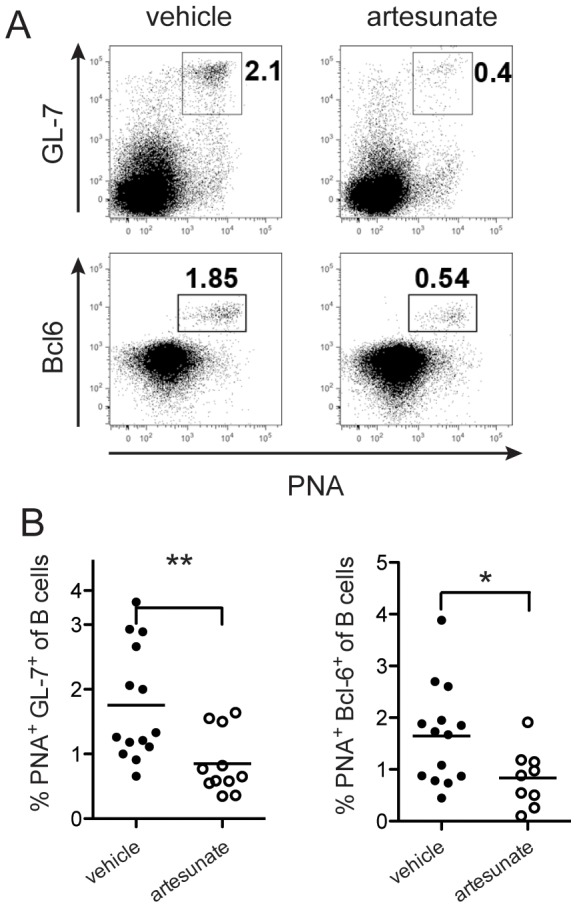
Effect of artesunate on GC B cells in established arthritis. Six-week-old K/BxN mice were treated with artesunate (100 mg/kg, twice/day) for 2 weeks. (**A**) Representative flow cytometric staining of GC B cells by GL-7 or Bcl-6 in combination with PNA. (**B**) Percent of GC B cells, each symbol represents one mouse (n = 12–14 per group); *  = p<0.05, **  = p<0.01.

**Figure 4:**
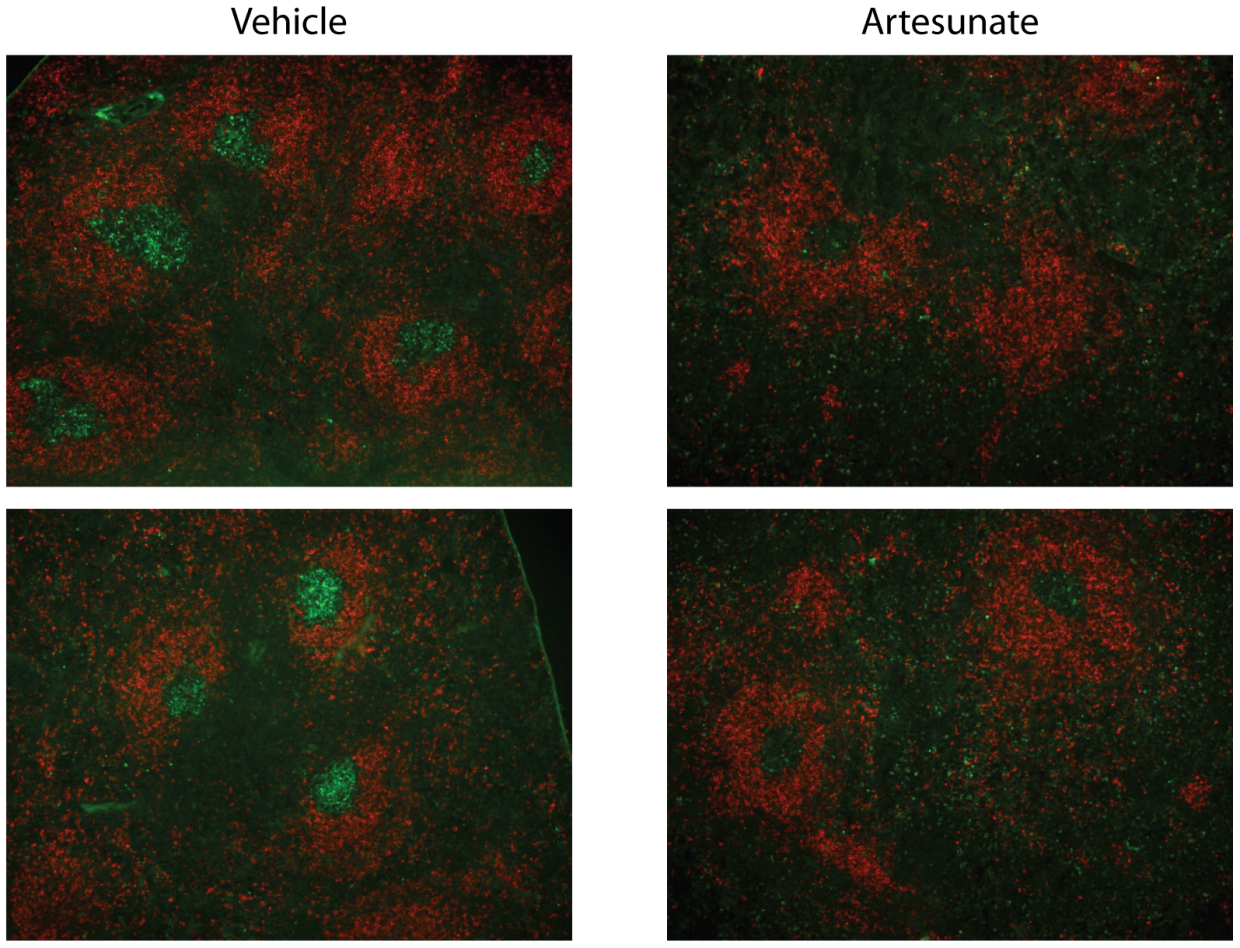
Immunohistochemistry of GCs after artesunate treatment. Six-week-old K/BxN mice were treated with artesunate for 2 weeks (100 mg/kg, twice/day). Cryosection of spleens were stained with PNA (green) and anti-IgD (red) to show GCs in B cell follicles and imaged at 100x magnification. Representative images from four mice per treatment.

To further test the efficacy of artesunate in a therapeutic setting, we treated 6-week old adult K/BxN mice with established arthritis. There was no improvement in ankle thickness or clinical index in mice treated with artesunate for 2 weeks to 2 months (data not shown). This finding was not surprising, as the half-life of IgG in mice is 5 to 9 days and the anti-GPI antibody titers in arthritic K/BxN mice are so high that the decay of autoantibodies was not fast enough during the period of treatment. Even B cell depletion therapy with rituximab does not reverse disease in this model [22]. This is because after arthritis has been established, cartilage erosion is too extensive to repair and no change in B cell function can reverse the joint damage [22]. Nevertheless, flow cytometric analysis showed that 2-week treatment of artesunate significantly decreased the proportion of GC B cells in K/BxN mice with established arthritis (Fig. 3A and 3B). We next examined the GCs by immunohistochemistry of frozen spleen sections. As shown in Fig. 4, there were many distinct GCs in the spleens of vehicle-treated K/BxN mice as revealed by PNA and anti-IgD staining. In contrast, GC B cells were significantly decreased in artesunate treated mice. Thus, in K/BxN mice with established arthritis, artesunate treatment could also suppress the GC response through abrogating GC B cells.

**Figure 5:**
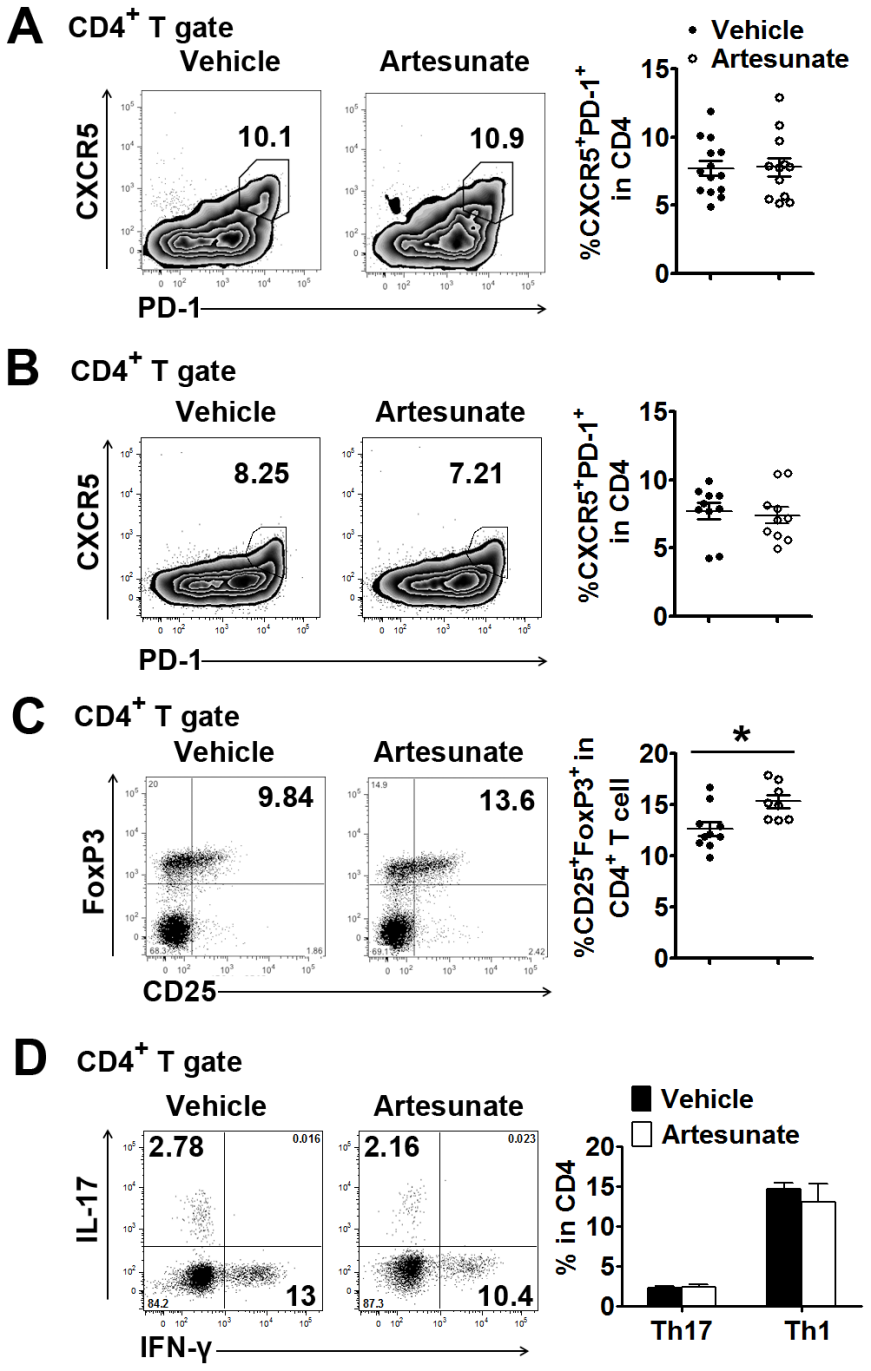
The effect of artesunate on various T cell susbsets. Six-week-old K/BxN mice were treated with artesunate for 2 weeks ((A), 100 mg/kg, twice/day) or 2 months ((B to D), 100 mg/kg, once/day). (**A** and **B**) Flow cytometry measuring surface PD-1 and CXCR5 expression in the CD4^+^ population. (**C**) Flow cytometry measuring surface CD25 and intracellular Foxp3 expression in the CD4^+^ population. (**D**) Intracellular staining of IL-17 and IFN-γ in the CD4^+^ population. (A to D) Left panel: representative flow cytometric results. Right panel: cumulative statistical results. For A to C, each symbol represents one mouse (n = 12-14 per group). For D, shown are mean ± SEM, n = 4 in each treated group; *  = p<0.05.

Flow cytometric analysis showed no effect on the Tfh cell percentage, by expression of PD-1 and CXCR5, in K/BxN mice treated with artesunate for two weeks (Fig. 5A). To further confirm the differential effects of artesunate on GC B cells and Tfh cells, we prolonged the treatment period from 2 weeks (2 injections/day) to 2 months (1 injection/day). Even in mice treated with artesunate for 2 months, the Tfh cells were still not affected when compared with vehicle treated mice (Fig 5B). Artemisinin analogues have been reported to induce Treg cell differentiation and suppress Th1 and Th17 cell differentiation [6], [10], [13], [16]. Therefore we analyzed these T cell subsets in K/BxN mice treated with artesunate. Artesunate treatment induced a slight but significant increase in the percentage of Foxp3^+^CD25^+^ Tregs in the spleens of K/BxN mice (Fig. 5C). There was no difference in Th1 or Th17 cells between vehicle- and artesunate-treated K/BxN mice (Fig. 5D).

### Artesunate diminishes GC B cells by inhibiting proliferation

**Figure 6:**
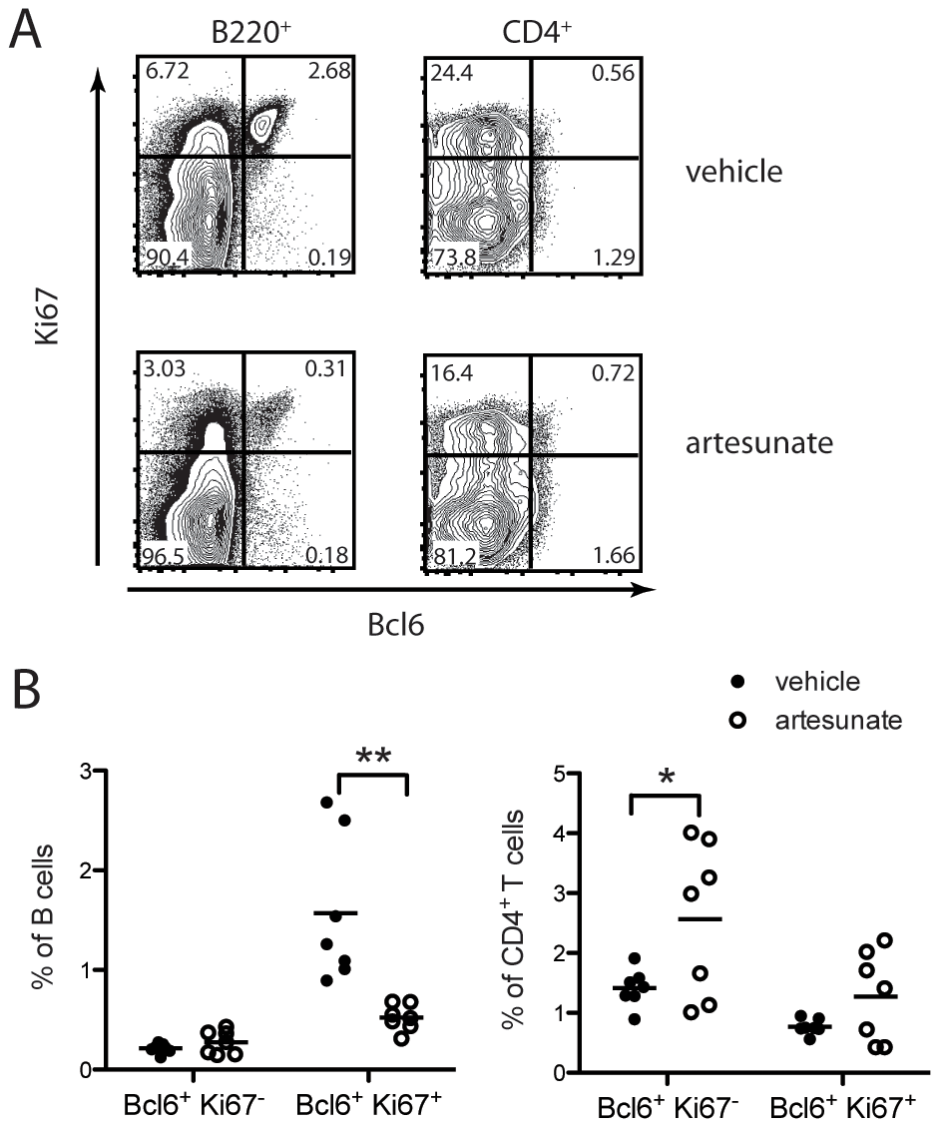
Proliferation of GC B cells during artesunate treatment. Six-week-old K/BxN mice were treated with vehicle or artesunate (100 mg/kg, twice/day) for 3 days. At the termination of treatment, splenocytes were stained with surface CD4, B220, and intracellular Bcl-6, and Ki-67. (**A**) Representative flow cytometric staining of Bcl-6 and Ki-67 in CD4^+^ and B220^+^ gates. (**B**) Cumulative statistic results. Shown are results from two experiments. Each symbol represents a mouse. *  = p<0.05, **  = p<0.01.

To further address the mechanism of artesunate to diminish GC B cells, we tested whether artesunate specifically targets proliferating GC B cells. As expected, GC B cells were highly proliferative and the vast majority expressed high level of Ki-67 (Fig. 6A). Artesunate treatment for 3 days significantly decreased the Ki-67^+^ GC B cells but not Ki-67^-^ GC B cells (Fig. 6B). There was a modest decrease of proliferating non-GC B cells (Ki-67^+^Bcl-6^-^) after artesunate treatment (5.7±0.9% in vehicle group and 4.0±0.8% in artesunate group). In contrast, Ki-67 expression was comparable in both Tfh cell and non-Tfh CD4^+^ T cells, confirming the minimal proliferation of this cell subset [27]. Artesunate treatment did not significantly change the percentage of Ki-67^+^ Tfh cells and may have even increased the percentage of Ki-67^-^ Tfh cells (Fig. 6B). These data suggest the proliferating GC B cells are preferentially more sensitive to artesunate treatment.

## Discussion

There is increasing evidence that artemisinin and its derivatives have immunosuppressive effects. In this study, we investigated the therapeutic effects of artesunate on arthritis in K/BxN mice. The well-understood etiology and pathogenesis of K/BxN mouse model allowed us to pinpoint the potential mechanisms underlying the immunosuppressive properties of artesunate. Our results show that artesunate treatment could prevent the onset of arthritis and inhibit anti-GPI antibody production, mediated by abolishing GC B cells. The ablation of GC B cells in established arthritis was as dramatic as in prophylactic settings. Furthermore, artesunate targeted specifically the proliferating GC B cells. In contrast, artesunate treatment did not affect Tfh cells that are specialized T helper subset to help GC B cells, nor did it influence Th1 and Th17 cell development.

Recent reports have shown that artemisinin analogues can inhibit proliferation of both T and B cells stimulated by mitogens [5], [14], [15]. Artesunate is more effective in suppressing the proliferation of B cells (IC50 = 0.989 µM) than T cells (IC50 = 4.58 µM). Additionally, artesunate was more potent than artemisinin (IC50 = 8.96 µM) and artemether (IC50 = 1.78 µM) to suppress B cell proliferation induced by LPS stimulation [14], [15]. However, most investigations to date have focused on their regulating the balance between pathogenic T cells and regulatory T cells, leaving the effects on humoral response unexplored. The GC is the microanatomical structure of secondary lymph organs during an immune response. It is the site of somatic hypermutation, affinity maturation, class switching that leads to the production of high affinity antibodies. Whereas naive B cells rarely divide, GC B cells are among the fastest dividing mammalian cells, with a cell-cycle time estimated at between 6 and 12 hrs (reviewed in [23]). It seems plausible that the more proliferative a population is, the more susceptible it is to artesunate, although other characteristics of GC B cells may underlie their high sensitivity to artesunate. Importantly, the default fate for a GC cell is to die by apoptosis. GC B cells express high levels of the death receptor Fas, lose expression of the antiapoptotic molecule Bcl-2, and, when placed in culture, die within a few hours (reviewed in [23]).

It has been shown that artemisinin analogues could induce Tregs in autoimmune models in vivo [6], [7], [12], [13]. In the current study, we also found that artesunate treatment induced a slight but significant increase in Treg cells in K/BxN mice. Treg cells play a suppressive role in many autoimmune diseases and can potentially affect various steps in the progression of disease. However, manipulation of Treg cells in the K/BxN model and collagen induced arthritis model did not affect the antibody titers [33], [34], suggesting that the effect of artesunate on GC B cells and antibody production is not secondary to its effect on Treg cells.

Innate inflammatory components are critical to mediate the downstream effector phase of pathogenesis and to amplify the local tissue injury. Previous reports have showed that artesunate can decrease the secretion of proinflammatory cytokines from TNF-α-stimulated human rheumatoid arthritis fibroblast-like synoviocytes [9]. When applied to collagen-induced arthritis in rats, artesunate showed promising therapeutic effects [10]. Accordingly, other artemisinin analogues also showed some anti-inflammatory properties in collagen-induced arthritis in DBA/1 mice [8]. However, it has been difficult to assess the direct vs. indirect effect of artemisinin analogues on the inflammatory response. Taking advantage of the serum transfer model of K/BxN mice, we were able to test the direct effect of artesunate on inflammatory phase, which is mediated solely by transferred autoantibodies and host innate immune cells without immunization and boosting. Our result showed that artesunate was far less effective in serum transfer-induced arthritis than in spontaneous K/BxN mice. Thus, our results strongly argue that artesunate primarily suppresses the adaptive immune activation, rather than the innate inflammatory responses, to treat the autoimmune arthritis. It would be informative to revisit these other models of autoimmunity to investigate whether the effect of artesunate acted at a similar phase.

## Conclusions

In summary, artemisinin analogues can affect multiple immune cell populations in autoimmune diseases. The relative contribution of these cells to pathogenesis is likely to be dependent on the particular model or disease and therefore artemisinin analogues can affect autoimmune diseases through multiple mechanisms. Our demonstration that GC B cells are very sensitive to artesunate treatment provides a new rationale for treating autoantibody-mediated autoimmune diseases.
